# Ferritinophagic Flux Activation in CT26 Cells Contributed to EMT Inhibition Induced by a Novel Iron Chelator, DpdtpA

**DOI:** 10.1155/2019/8753413

**Published:** 2019-06-20

**Authors:** Yanjie Sun, Cuiping Li, Jiankang Feng, Yongli Li, Xinbo Zhai, Lei Zhang, Changzheng Li

**Affiliations:** ^1^Department of Molecular Biology and Biochemistry, Xinxiang Medical University, Xinxiang, Henan 453003, China; ^2^Experimental Teaching Center of Biology and Basic Medical Sciences, Sanquan College of Xinxiang Medical University, Xinxiang, Henan 453003, China; ^3^Department of Histology and Embryology, Sanquan College of Xinxiang Medical University, Xinxiang, Henan 453003, China; ^4^Laboratory of Molecular Medicine, Xinxiang Medical University, Xinxiang, Henan 453003, China

## Abstract

Epithelial-mesenchymal transition (EMT) contributes to metastasis and drug resistance; inhibition of EMT may attenuate metastasis and drug resistance. It has been demonstrated that ferritinophagy involves the process of many diseases; however, the relationship between EMT and ferritinophagy was not fully established. Some iron chelators show the ability to inhibit EMT, but whether ferritinophagy plays a role in EMT is largely unknown. To this end, we investigated the effect of a novel iron chelator, DpdtpA (2,2 ′-di-pyridylketone dithiocarbamate propionic acid), on EMT in the CT26 cell line. The DpdtpA displayed excellent antitumor (IC_50_ = 1.5 ± 0.2 *μ*M), leading to ROS production and apoptosis occurrence. Moreover, the ROS production correlated with ferritin degradation. The upregulation of LC3-II and NCOA4 from immunofluorescence and Western blotting analysis revealed that the occurrence of ferritinophagy contributed to ROS production. Furthermore, DpdtpA could induce an alteration both in morphology and in epithelial-mesenchymal markers, displaying significant EMT inhibition. The correlation analysis revealed that DpdtpA-induced ferritinophagy contributed to the EMT inhibition, implying that NCOA4 involved EMT process, which was firstly reported. To reinforce this concept, the ferritinophagic flux (NCOA4/ferritin) in either treated by TGF-*β*1 or combined with DpdtpA was determined. The results indicated that activating ferritinophagic flux would enhance ROS production which accordingly suppressed EMT or implementing the EMT suppression seemed to be through “fighting fire with fire” strategy. Taken together, our data demonstrated that ferritinophagic flux was a dominating driving force in EMT proceeding, and the new finding definitely will enrich our knowledge of ferritinophagy in EMT process.

## 1. Introduction

Epithelial-to-mesenchymal transition (EMT) is a cellular process allowing epithelial cells to undergo several biochemical alterations that permit a polarized epithelium switches to a highly invasive mesenchymal phenotype [1], accordingly suppression of epithelial markers; upregulation of mesenchymal markers occur [2, 3]. It has been demonstrated that the transforming growth factor (TGF), receptor tyrosine kinase (RTK), Wnt, Notch, hedgehog, hippo, cytokine, and nuclear receptor pathways have all been implicated in the onset of EMT [4, 5]. In addition, microRNAs (miRNAs), hypoxia, and the generated reactive oxygen species (ROS) can also induce EMT [6]. EMT is considered as a crucial event in cancer metastasis. During metastasis, the malignant cells spread from the primary tumor to distant site, which causes failure of vital organs, consequently leading to the death of patients. In addition, accompanied with metastasis, the cells acquire an ability to resist conventional treatments [7]. Therefore, insight into the cellular, molecular mechanism of EMT is required in order to develop new diagnostic and therapeutic strategies to prevent and treat metastases.

EMT is considered as a driving force in tumor progression, while compelling evidence reveals reactive oxygen species (ROS) as crucial conspirators in EMT engagement [8]. ROS are radicals, ions, or molecules that own a single unpaired electron in their outermost shell of electrons. They are constantly generated inside cells by a serial of dedicated enzyme complexes or as by-products of redox reactions, such as mitochondrial respiration [9–11]. ROS act as signaling molecules and are finely modulated and responsive to a wide array of environmental cues [10]. In addition, the degradation of iron-containing macromolecules (ferritin and mitochondrial components) or endocytosed erythrocytes (by macrophages) in lysosomes contributes large amounts of iron that can be reduced to Fe(II) in reducing lysosomal environment. Fe(II) either in lysosome or in labile iron pool (LIP) is known to catalyze Fenton reaction, yielding extremely reactive hydroxyl radicals [12]. As mentioned above, accumulating evidence shows that ROS are involved in EMT transition [13–17]; however, the functions of ROS in the processes of EMT remain unclear.

Since redox regulates epithelial-mesenchymal transition of tumor cell, scavenging ROS favors restoration of MET (mesenchymal-to-epithelial transition), which may efficiently slow dissemination of tumor cells [18, 19]. A lot of compounds either from natural products or artificially synthesized exhibit the ability in EMT reversion; different signal pathway was proposed [20–23]. Recently, iron depletion has been discovered to suppress tumor growth and EMT phenotypes via upregulation of the N-Myc downstream-regulated gene 2 (NDRG2) [24–27].

Labile iron pool (LIP) is regulated by ferritin, a highly conserved iron storage protein which is composed of two subunits, H-ferritin and L-ferritin, and the twelve pairs of subunits binding head to foot form the 24 subunit ferritin cages [28]. When the iron level in the cell is low, ferritin is degraded allowing the release of iron for use by the cell; therefore, ferritin plays an important role in cellular redox modulation. Iron chelator can induce ferritin degradation through ubiquitination or autophagy; the former occurs in proteasomes, latter in lysosomes. The worn-out proteins or organelle generally is degraded in lysosomes, and microtubule-associated protein light chain 3 (LC3) is involved in the proteolytic process. During autophagy, the cytosolic form of LC3 (LC3-I) conjugates to phosphatidylethanolamine to form LC3-phosphatidylethanolamine conjugate (LC3-II), which is recruited to autophagosomal membranes that engulf the damaged proteins. It has shown that when ferritin degrades in lysosome, nuclear receptor coactivator 4 (NCOA4) is required for ferritin delivery to autophagosome; the proteolytic process is termed ferritinophagy [29]. Currently, iron chelator, DFO and DpdtC, has been reported to own the ability in ferritinophagy induction [29, 30], but whether the iron chelator induced EMT reversion through ferritinophagy remains to be determined. In the present study, we firstly reported that activating ferritinophagic flux (NCOA4/ferritin) could inhibit EMT in the DpdtpA-treated CT26 cell, indicating that NCOA4 also involves in EMT process. Although the effect of autophagy on EMT is contradictory in different literature [31], and the role of ferritinophagy in EMT is not fully determined, therefore the results from this study will definitely enrich our knowledge in EMT transition.

## 2. Results

### 2.1. The Antiproliferative Action of the DpdtpA Was ROS Dependent

**Figure 1:**
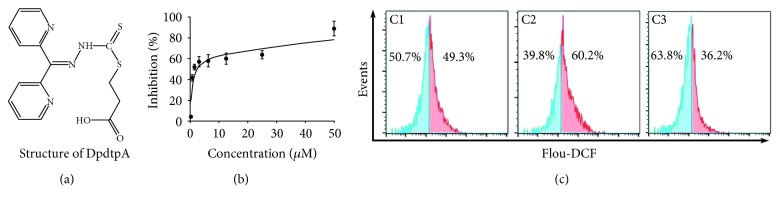
DpdtpA-induced growth inhibition involved ROS generation. (a) Structure of DpdtpA; (b) antiproliferative effect of DpdtpA on the CT26 cells; (c) DpdtpA induced ROS production: C1, 0.7% DMSO; C2, 0.78 *μ*M DpdtpA; C3, 0.78 *μ*M DpdtpA+NAC (1.5 mM). The data from MTT were from five measurements, and ROS assays were conducted twice.

2,2 ′-Di-pyridineketone hydrazone dithiocarbamate propionic acid (DpdtpA, Figure 1(a)) displayed significant proliferative inhibition against hepatoma carcinoma cell line in previous study, and its copper complex owned enhanced proliferative inhibition compared to DpdtpA; however, this did not correlate with ROS production [32]. To determine that whether the correlation between ROS production and growth inhibition was cell line dependent, we firstly evaluated the effect of DpdtpA on the proliferation of the CT26 cells. The dose-response curve is depicted in Figure 1(b). As expected, DpdtpA had significant growth inhibition for CT26 cells (IC_50_: 1.5 ± 0.2 *μ*M). Next, the cellular ROS level was measured by flow cytometry as described previously [33]. As shown in Figure 1(c), the populations in higher fluorescence intensities significantly increased by ~10% after the exposure of DpdtpA to the cells for 24 h, but the addition of NAC, a ROS scavenger, significantly decreased ROS production (~23%), hinting that the antiproliferative action involved ROS production. However, whether ROS production contributed to the growth inhibition needed to be further determined; thus, the growth inhibition in the presence of NAC was further assessed. As shown in Figure S1, the addition of NAC (0.15 mM) could attenuate the inhibitory ability of DpdtpA on the proliferation of the CT26 cell, indicating that ROS production played a role in the growth inhibition.

### 2.2. DpdtpA-Induced Growth Inhibition Involved Apoptosis

**Figure 2:**
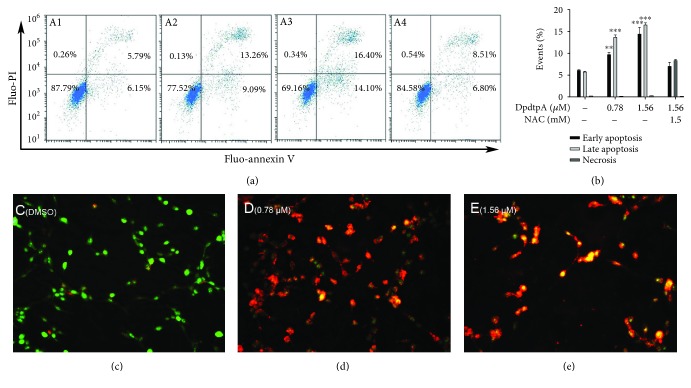
A DpdtpA induced apoptosis in the CT26 cell after 24 h posttreatment as detected by flow cytometry and EtBr/AO staining assay. Flow cytometry analysis: (A1) DMSO; (A2) 0.78 *μ*M DpdtpA; (A3) 1.56 *μ*M DpdtpA; (A4) 1.56 *μ*M DpdtpA+NAC (1.5 mM). (b) Quantification analysis of apoptosis and necrosis induced by DpdtpA. The data were from two measurements. Microscopical analysis by EtBr/AO stains: (c) DMSO; (d) 0.78 *μ*M DpdtpA; (e) 1.56 *μ*M DpdtpA. Green, orange, and red fluorescence indicates live, apoptotic, and dead cells, respectively. Images were captured by fluorescence microscope (Nikon ECLIPSE TiE) at ×10 magnification. AO: acridine orange; EtBr: ethidium bromide. The measurements were performed thrice from different field of view (AO/EtBr stains); the quantification analysis of apoptosis by flow cytometry was from two measurements.

Since ROS production involved the growth inhibition induced by DpdtpA, the apoptosis might occur. To determine the correlation, the apoptotic populations at early and late stages were evaluated via flow cytometry after annexin V/propidium iodide (PI) staining, which measures externalization of phosphatidylserine on the cell surface of apoptotic cells specifically.  Figure 2 showed that DpdtpA-induced early apoptosis and late apoptosis were in a concentration-dependent manner (Figure 2, A1-A3, from 11.94 to 30.5%, DpdtpA-induced early apoptosis and late apoptosis compared to control had statistical significance, *p* < 0.05 or 0.01); however, the apoptosis induction could be attenuated by addition of NAC (Figure 2, A4), indicating that ROS production played a role in the growth inhibition of DpdtpA. The quantification analysis of each group in apoptosis and necrosis is presented in Figure 2(b). In addition, detection of cell apoptosis using AO/EB is an additional method; thus, assay of apoptosis engagement via AO/EtBr stains was also conducted by the aid of fluorescence microscope [33, 34]. As shown in Figures 2(c)–2(e), live cells appeared uniformly green and had intact membrane, whereas early apoptotic cells and late apoptotic cells appeared as bright green and orange, respectively; necrotic cells appeared as red. Those supported that DpdtpA-induced growth inhibition involved apoptosis.

### 2.3. DpdtpA Induced Ferritin Degradation

**Figure 3:**
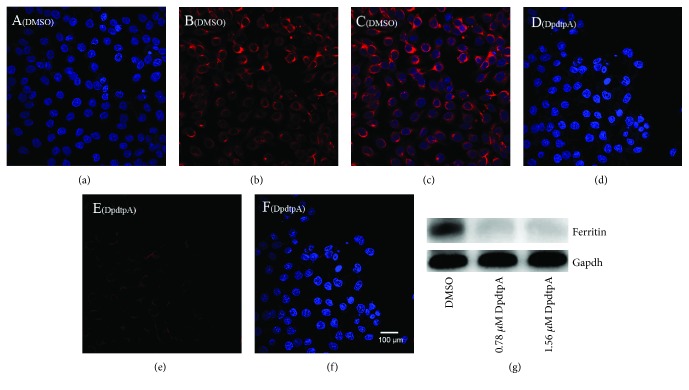
DpdtpA induced ferritin degradation. The nuclei stained by DAPI in blue, ferritin labeled in red. (a-c) Control group (DMSO): (a) nuclei in blue; (b) ferritin in red; (c) merge of nuclei and ferritin. (d-f) DpdtpA-treated CT26 cells: (d) nuclei in blue; (e) ferritin in red; (f) merge of nuclei and ferritin. The measurements were performed thrice. (g) Western blotting analysis. Scale bar: 100 *μ*m.

As described above, DpdtpA-induced growth inhibition involved ROS production; thus, the potential sites of ROS production were further explored. As well documented, ferritin degradation triggers Fenton reaction that is an important contributor in ROS production; therefore, the level of ferritin was determined; Figure 3(g) showed the status of ferritin based on Western blotting analysis. It was clear that DpdtpA indeed induced ferritin downregulation, which hinted that the ROS production may attribute to ferritin degradation. Similarly, the additional evidence from immunofluorescence analysis further supported ferritin downregulation after DpdtpA treatment as the red fluorescence of ferritin was significantly decreased (Figure 3(b)) compared to that of control (Figure 3(e)); those indicated that the growth inhibition induced by DpdtpA involved ferritin degradation that may contribute the ROS production.

### 2.4. DpdtpA Exposure Induced an Occurrence of Ferritinophagy

**Figure 4:**
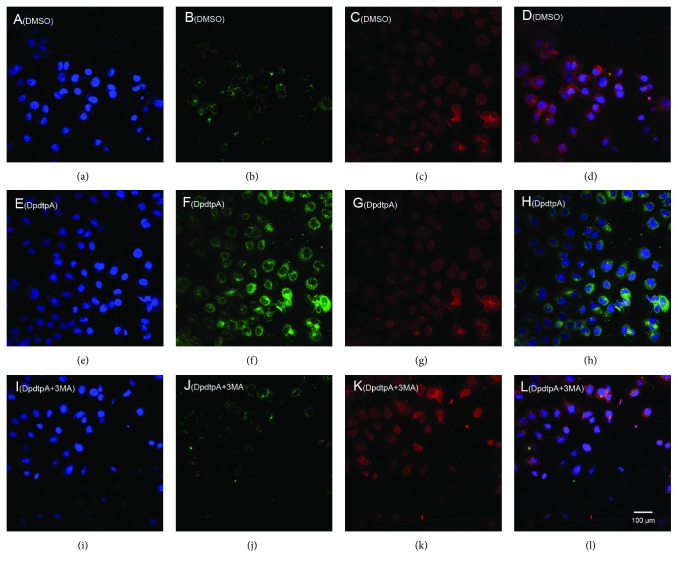
DpdtpA induced ferritin autophagy (ferritinophagy). The nuclei stained by DAPI in blue, ferritin labeled in red; LC3 labeled in green. (a-d) Control group: (a) nuclei in blue; (b) ferritin in red; (c) LC3 in green; (d) merge of ferritin with LC3. (e-h) DpdtpA-treated group: (e) nuclei in blue; (f) ferritin in red; (g) LC3 in green; (h) merge of ferritin with LC3. (i-l) DpdtpA combined with 3-MA group: (i) nuclei in blue; (j) ferritin in red; (k) LC3 in green; (l) merge of ferritin with LC3. The experiments were performed thrice. Scale bar: 100 *μ*m.

**Figure 5:**
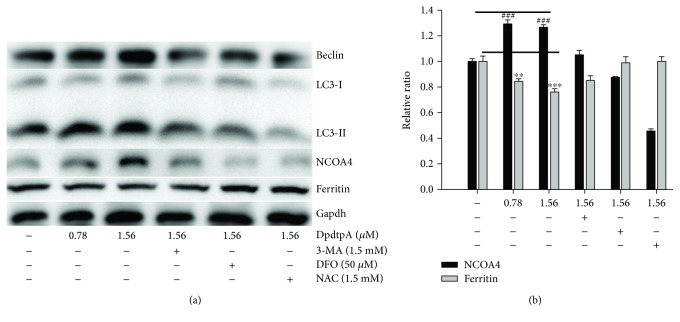
DpdtpA exposure resulted in alterations of ferritinophagy and autophagy proteins. (a) Western blotting analysis of autophagic and ferritinophagic proteins; (b) the quantitative comparisons of the proteins from (a). The quantification analysis of NCOA4 and ferritin was from two experiments. The condition was as indicated in the figure (^∗∗^ *p* < 0.05; ^∗∗∗,###^ *p* < 0.01).

Next, we determined the site of ferritin degradation; it may occur in proteasomes. To this end, the levels of ferritin either in the condition of DpdtpA treatment alone or its combination with a proteasomes inhibitor, MG132, were determined. Beyond our expectation, the addition of MG132 did not attenuate the ferritin downregulation induced by DpdtpA (Figure S2), indicating that the ferritin degradation was not through the ubiquitination pathway. Thus, the proteolysis of ferritin in lysosomes could be further considered. To test the hypothesis, the changes of cellular ferritin and autophagic marker, LC3, were monitored via immunofluorescence technique. As shown in Figure 4, DpdtpA exposure resulted in upregulated LC3 (green in Figure 4(g)) and downregulated ferritin (red in Figure 4(f)) compared to control (Figures 4(b) and 4(c)), hinting that the ferritin degradation might be through autophagic proteolysis. To corroborate the above hint, the 3-MA, an inhibitor in the formation of autophagic vacuole, was added during the exposure of DpdtpA to the CT26 cells. As expected, the ferritin degradation was significantly attenuated (Figure 4(j)), and the level of ferritin and LC3 was restored to that of control (Figures 4(d) and 4(k)), further supporting that the downregulated ferritin was due to autophagic degradation. In addition, since ferritin degradation occurs through autophagy, the ferritinophagy may occur, which requires a specific carrier, NCOA4 for ferritin degradation; thus, cellular levels of NCOA4, ferritin, and autophagy-related proteins before and after DpdtpA exposure were determined by Western blotting. As shown in Figure 5, the ferritin was decreased with increased DpdtpA, but the autophagic markers (LC3-II, beclin) and NCOA4 were increased with increased DpdtpA; however, the addition of 3-MA or DFO as well as NAC could markedly attenuate those upregulated proteins. The quantitative comparisons of ferritin and NCOA4 are shown in Figure 5(b); moreover, similar analyses of LC3-II and beclin were performed in Figure S3. Those clearly indicated that DpdtpA indeed induced an occurrence of ferritinophagy, acting as other ferritinophagy-inducing agents [29, 30]. To further support ferritinophagy occurrence, the total iron content before and after DpdtpA exposure to the CT26 cells was determined by atomic absorbance spectrometry. As shown in Figure S4, DpdtpA treatment resulted in markedly decrease of iron abundance, in accordance with results from immunofluorescence and Western blotting analysis.  Figure S5 showed that DpdtpA induced formation of massive autophagic vacuoles, which could be attenuated by 3-MA, or DFO as well as NAC, indicating that ferritin degradation correlated to autophagy that contributed to ROS production.

### 2.5. The DpdtpA Induced EMT Reversal

**Figure 6:**
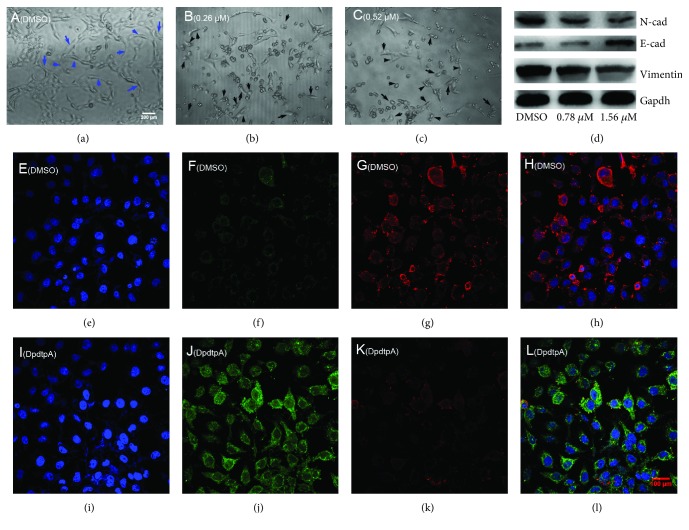
DpdtpA induced alteration in morphology correlated with EMT modulation. (a-c) Alteration in morphology treated by DpdtpA for 48 h at indicated concentration; the blue arrow: spindle-shaped cells, black arrow: retracted and rounded cells; (a) 0.7% DMSO; (b) 0.26 *μ*M DpdtpA; (c) 0.52 *μ*M DpdtpA; objective size: 20 × 10, scale bar: 200 *μ*m. (d) Western blotting analysis. (e-l) Immunofluorescence analysis of epithelial-mesenchymal markers. (e-h) (0.7% DMSO): (e) nuclei in blue; (f) E-cadherin in green; (g) vimentin in red; (h) merge of nuclei, E-cadherin, and vimentin in the DMSO group. (i-l) DpdtpA-treated group: (i) nuclei in blue; (j) E-cadherin in green; (k) vimentin in red; (l) merge of nuclei, E-cadherin and vimentin in the DpdtpA-treated group. The measurements were performed thrice from different field of view. Objective size: 40 × 10 (fluorescence), scale bar: 100 *μ*m.

DpdtpA displayed significant growth inhibition for CT26 cells; the effect of it on cellular morphology was further determined. As shown in Figures 6(a)–6(c), the exposure of DpdtpA led to evident alteration in morphology, which encouraged us to consider whether it affected EMT transformation, favoring metastasis inhibition of cancers [35]. Thus, the molecular alterations in epithelial-mesenchymal markers were investigated before and after the exposure of DpdtpA to CT26 cells. The immunofluorescence is widely used to label an interested protein; to this end, CT26 cells were stained by fluorescence antibody after treatment of DpdtpA. As shown in Figure 6, the green fluorescence of E-cadherin was increased (Figures 6(f) and 6(j)), while the red fluorescence which represented vimentin was decreased (Figures 6(g) and 6(k)). The merged photographs (Figures 6(h) and 6(l)) clearly showed the alterations in E-cadherin and vimentin; the proportion of red fluorescence (vimentin) in Figure 6(h) was much higher than that in Figure 6(l), indicating that DpdtpA could inhibit the EMT. The supporting evidence from Western blotting also demonstrated that DpdtpA could downregulate mesenchymal marker, vimentin, and N-cadherin, contrarily upregulated epithelial marker, E-cadherin (Figure 6(d)), in accordance with that from immunofluorescence analysis, supporting the inhibitory effect of DpdtpA on EMT transformation.

### 2.6. DpdtpA Suppressed TGF-*β*1-Induced EMT through the Ferritinophagy Pathway

**Figure 7:**
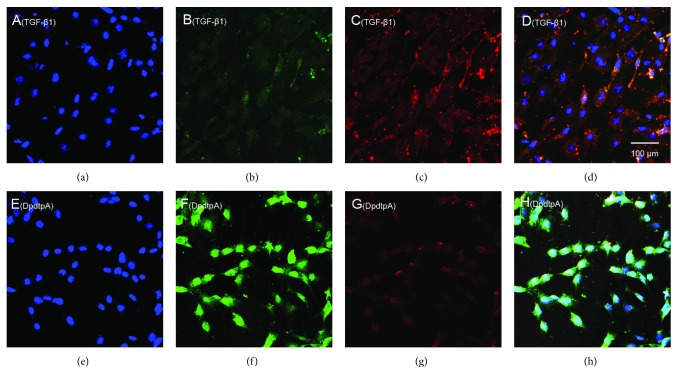
DpdtpA resisted TGF-*β*1-induced EMT. (a-d) (TGF-*β*1-treated group): (a) nuclei stained by DAPI in blue (DMSO); (b) E-cadherin in green; (c) vimentin in red; (d) merge of nuclei, E-cadherin, and vimentin. (e-h) (TGF-*β*1 treatment plus DpdtpA): (e) nuclei in blue; (f) E-cadherin in green; (g) vimentin in red; (h) merge of nuclei, E-cadherin, and vimentin. The measurements were performed thrice from different field of view. Objective size: 40 × 10, scale bar: 100 *μ*m.

As mentioned above, DpdtpA could modulate expressions of epithelial-mesenchymal markers, owning the ability to suppress EMT. To validate the ability of DpdtpA, a model that was undergoing EMT needed to be created. TGF-*β*1 is the most powerful EMT inducer; thus, the CT26 cells were pretreated with TGF-*β*1 for 48 h, which resulted in obviously morphological alteration. As shown in Figure S6, the CT26 cells became more spindle-shaped, fibroblast-like cells, and these morphological characteristics were considered cells undergoing the EMT [36]. Next, we examined the effect of DpdtpA on EMT induced by TGF-*β*1. Immunofluorescence analysis revealed that TGF-*β*1 markedly reduced the level of E-cadherin in the CT26 cells (Figure 7(b)); however, the reduction in the E-cadherin level was markedly attenuated after DpdtpA treatment (compared Figure 7(b) with Figure 7(f)). As the E-cadherin increased, the upregulated vimentin was significantly suppressed by DpdtpA (Figures 7(c) and 7(g)); the merged photographs (Figures 7(d) and 7(h)) clearly showed the alterations of epithelial-mesenchymal transformation before and after treatment of DpdtpA, corroborating that DpdtpA was able to resist TGF-*β*1-induced EMT.

**Figure 8:**
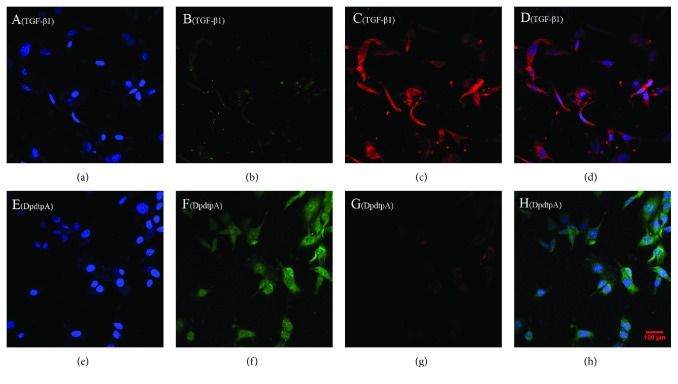
DpdtpA induced ferritinophagy in the presence of TGF-*β*1. The nuclei stained by DAPI in blue, NCOA4 in green, and ferritin in red. (a-d) (0.7% DMSO, TGF-*β*1 treatment only): (a) nuclei in blue; (b) NCOA4 in green; (c) ferritin in red; (d) merge of nuclei, ferritin, and NCOA4. (e)-(h) TGF-*β*1 combined with DpdtpA: (e) nuclei in blue; (f) NCOA4 in green; (g) ferritin in red; (h) merge of nuclei, ferritin, and NCOA4. Objective size: 40 × 10, scale bar: 100 *μ*m. The measurements were performed thrice from different field of view.

Next, we further questioned whether DpdtpA still induced ferritinophagy in the presence of TGF-*β*1. To this end, the levels of cellular NCOA4 and ferritin were determined by immunofluorescence. As shown in Figure 8, DpdtpA treatment markedly increased NCOA4 expression and attenuated ferritin expression, indicating that DpdtpA-suppressed TGF-*β*1-inhibited EMT involved ferritinophagy.

**Figure 9:**
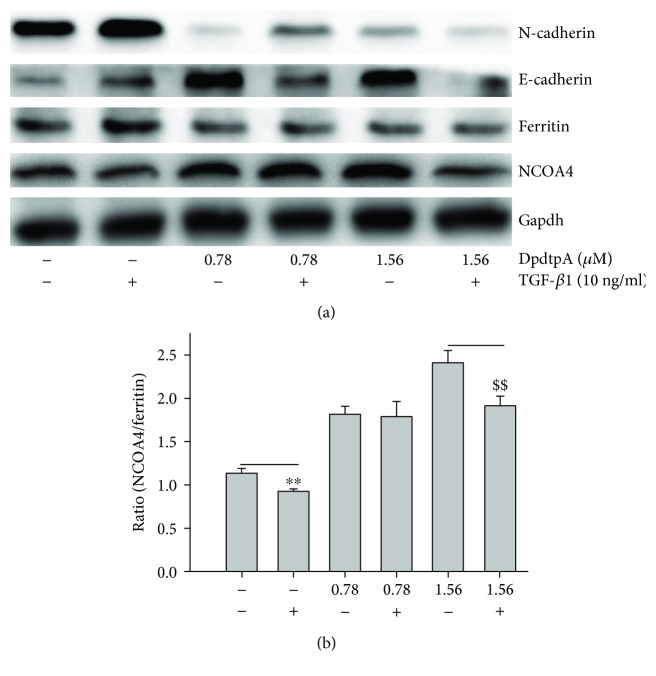
Ferritinophagic flux played an important role in EMT process. (a) The alterations in ferritinophagy and EMT-related proteins when either TGF-*β*1 or combined with DpdtpA treatment; (b) quantitative analysis of the ferritinophagic flux in the investigated condition. The quantification analysis of ferritinophagic flux was from three experiments (^∗∗^^, $$^ *p* < 0.05; one-way ANOVA).

Since DpdtpA induced both EMT inhibition and ferritinophagy, we speculated there might be a correlation between the two events. Thus, we introduced a term, ferritinophagic flux, that was defined as the ratio of NCOA4/ferritin and observed how ferritinophagic flux influences EMT status. As shown in Figure 9, TGF-*β*1 boosted EMT (upregulated vimentin and downregulated E-cadherin) through lowering ferritinophagic flux, contrarily DpdtpA inhibited EMT (upregulated E-cadherin, downregulated vimentin) through elevating ferritinophagic flux (Figure 9(a)). Therefore, the level of ferritinophagic flux was an important index for EMT status. To further support the hypothesis, a small interfering RNA was used to knock down the ferritinophagy-specific cargo, NCOA4, which resulted in downregulation of E-cadherin and upregulation of vimentin (Figure S7), suggesting that NCOA4 was involved in EMT transformation. To the best of our knowledge, the involvement of NCOA4 in EMT was first reported in the present study.

### 2.7. ROS Production in Lysosomes Led to Alteration of Permeability of Lysosomal Membrane of the CT26 Cell

**Figure 10:**
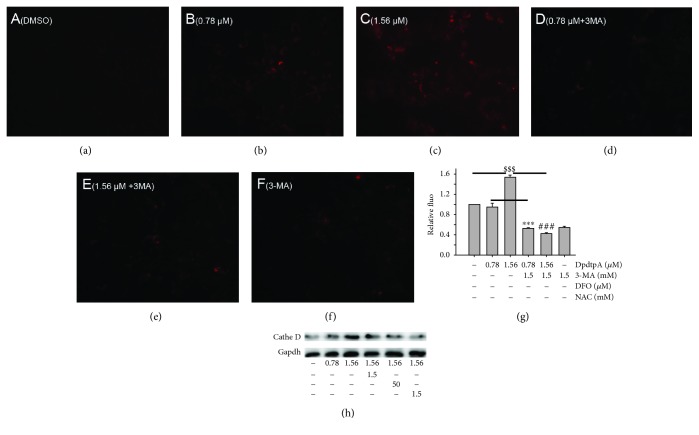
DpdtpA induced alteration in lysosomal membrane permeability and release of cathepsin D. (a) DMSO; (b) 0.78 *μ*M DpdtpA; (c) 1.56 *μ*M DpdtpA; (d) 0.78 *μ*M DpdtpA+3-MA (5 mM); (e) 1.56 *μ*M DpdtpA+3-MA (5 mM); (f) 3-MA (5 mM); (g) quantitative analysis of alteration in fluorescence after treated by either DpdtpA or combined with 3-MA; (h) DpdtpA induced cathepsin D release. The quantification analysis of intensity of red fluorescence was from three measurements. The Western blots were performed twice (^∗∗∗^^, ###, $$$^ *p* < 0.01).

As described in Section 2.1, the cellular ROS were increased when DpdtpA was exposed to the CT26 cells. It was conceivable that the intracellular ROS at least partially were generated from lysosomes due to occurrence of ferritinophagy that may trigger Fenton-like reaction [37]. To test the speculation, the lysosomal membrane of permeability (LMP) was determined as described previously [38]. As shown in Figure 10, the lysosomotropic dye, LysoTracker Red was accumulated in lysosomes in a concentration-dependent manner (Figures 10(a)–10(c)), but addition of 3-MA drastically attenuated the accumulation (Figure 10(e)), in consistent with results from observation of ferritinophagy. In addition, addition of NAC and DFO (iron chelator) also markedly decreased the accumulation of the dye (Figures 10(d) and 10(f)); the quantitative analysis is shown in Figure 10(g). Those indicated that ROS production indeed occurred in lysosomes (Figure 10). Owing to the change of LMP, the hydrolase in lysosome may be released. Therefore, a protease cathepsin in lysosome was determined. As shown in Figure 10(h), more cathepsin D was presented in cytoplasm during DpdtpA treatment, but the level of cathepsin D could be attenuated by 3-MA, NAC, and DFO, supporting that ferritinophagy triggered Fenton reaction was a critical contributor in ROS production. In addition, the data from flow cytometry revealed that the DpdtpA suppressed TGF-*β*1-induced EMT seemed to be through producing more ROS (Figure S8). To gain more evidence, the levels of EMT-related proteins were further determined in the absence or presence of NAC. As shown in Figure S9, the addition of NAC could attenuate the EMT inhibition induced by DpdtpA, supporting that the EMT inhibition seemed to be achieved through “fighting fire with fire” strategy.

## 3. Discussion

Cancer metastasis is responsible for approximately 90% of all cancer-related deaths. Certain patients may benefit from resection, though mostly transiently for lack of clinical marker for surgeon due to cancer metastasis [39]; therefore, effective means are needed to suppress invasion and metastasis. To this end, understanding the underlying details of metastasis in a molecular level is required. It has been demonstrated that epithelial-mesenchymal transition (EMT) involves metastatic process, and EMT is a key metastasis-promoting step in many cancers [40]; thus, inhibiting EMT is an alternative option in cancer therapy. The occurrence of EMT results in cytoskeleton reorganization, loss of cell-cell adhesion molecules, and transformation to mesenchymal cell phenotype, which endows the cells with an invasive ability [41]; the alterations in abundance of E-cadherin, N-cadherin, and vimentin determine EMT status. A number of transcription factors are known to be involved in the regulation of EMT, such as ZEB 1 and ZEB 2, snail, slug, and twist [42]. In addition, growth factors and cytokines, ECM components, Wnt proteins, hypoxia, ROS, and mechanical stress trigger EMT [43]. Recently, the effect of ferritin heavy chain (FHC) on EMT has received widely attention. Karicheva et al. reported that oxidant-induced autophagy and ferritin degradation contributed to epithelial-mesenchymal transition through lysosomal iron in A549 cell line [16]. Zhang et al. revealed that FHC played a critical role in TGF-*β*1-induced EMT in AML-12 cells [44]. Aversa et al. showed that FHC silencing caused EMT in MCF-7 and H460 cell lines through the CXCR4/CXCL12 axis [45]. Meanwhile, those studies also demonstrated that ROS production stemmed from ferritin degradation (downregulation) had a role in EMT process. It is well documented that ferritin degradation may occur either in proteasomes through ubiquitination or in lysosomes via ferritinophagy; however, distinct route for its degradation (or regulation) in EMT process received lesser concern. Iron chelator induced ferritin degradation has been reported in several studies [27, 46]; some iron chelator can inhibit EMT [27], but whether NCOA4-mediated ferritinophagy engages EMT modulation remains unclear. In the present study, we presented a new insight into the role of ferritinophagic flux in EMT process. DpdtpA, a dithiocarbamate derivative, exhibited significant growth inhibition against the CT26 cells (Figure 1), which involved ROS-dependent apoptosis induction (Figure 2). To gain insight into the mechanism in ROS production, the level of ferritin before and after DpdtpA treatment was investigated, and a downregulation of ferritin both from immunofluorescence and Western blot was observed, hinting the ROS production might stem from ferritin degradation (Figure 3). To determine whether the ferritin degradation occurred in proteasomes, a proteasome inhibitor, MG132 was employed; as shown in Figure S2, the inhibitor did not attenuate ferritin decrease, indicating that DpdtpA-induced ferritin degradation was not through ubiquitination. Thus, lysosomal proteolysis could be considered. To support the hypothesis, the alterations in autophagic marker, LC3 and ferritinophagy-specific cargo, and NCOA4 were investigated; the data clearly showed that DpdtpA induced an occurrence of ferritinophagy as DpdtC acted (Figures 4 and 5) [30], hinting that ROS production may stem from lysosomal iron that triggered Fenton reaction. In addition, DpdtpA could also induce morphological change of the CT26 cells (Figure 6), which prompted us to consider that the DpdtpA might also affect EMT. To confirm the effect of DpdtpA on EMT, the levels of epithelial and mesenchymal marker after DpdtpA treatment were determined (Figure 6). As expected, the upregulated E-cadherin and downregulated vimentin clearly demonstrated that DpdtpA inhibited EMT. Furthermore, additional evidence from the EMT model induced by TGF-*β*1 validated the ability of DpdtpA [36], because the DpdtpA treatment resulted in an increase of E-cadherin and a decrease of vimentin (Figure 7), in accordance with other iron chelator acted [27].

It was interesting that the EMT inhibition was accompanied by upregulation of LC3 and NCOA4 and downregulation of ferritin (Figure 8), which hinted that there was a correlation between EMT and ferritin degradation. Thus, the term, ferritinophagic flux, was used to describe the correlation. As shown in Figure 9(b), TGF-*β*1 triggered EMT through attenuating ferritinophagic flux, but DpdtpA inhibited EMT through increasing ferritinophagic flux, indicating that ferritinophagic flux determined the status of EMT. To support the hypothesis, a small interfering RNA of NCOA4 was used to knockdown the ferritinophagy-specific cargo, which resulted in both downregulation of E-cadherin and upregulation of vimentin, supporting that NCOA4 was involved in EMT process. Our finding suggested that ferritinophagic flux was also a dominant driving force in EMT process, which firstly described that NCOA4 involved EMT transformation. Similar result was found in other cell lines (the results will be reported later). Currently, NCOA4 function differently depends on the cancer context, while robust evidence for the role of ferritinophagy in tumorigenesis is lacking [40]; here, we presented that NCOA4-mediated ferritinophagy contributed to EMT inhibition, which may enrich our knowledge for NCOA4 in tumor development.

Owing to occurrence of ferritinophagy, the abundance of lysosomal iron should be increased; oxidative stress and cell death may come up [47]. The consequence of ferritinophagy inevitably led to lysosomal destruction; Figure 10 showed that more LysoTracker Red dyes were accumulated in lysosomes after DpdtpA treatment, but this accumulation could be attenuated by addition of 3-MA, in accordance with the observation reported previously [30], indicating that the damage of lysosomal membrane (or integrity) was due to occurrence of ferritinophagy. It was imagined that the ferric iron liberated from digested ferritin would reduce further by the endosomal ferrireductase Steap3 in the acidified lysosome [48]; the resulting ferrous ion triggered Fenton reaction. To confirm that the lysosomal ROS production contributed to EMT transformation, the ROS production in either stimulating by TGF-*β*1 or combined with DpdtpA was investigated. TGF-*β*1 indeed induced rising of ROS in the CT26 cells, in accordance with reported previously [16]. It was more interesting that ROS production induced by DpdtpA exceeded that triggered by TGF-*β*1, seeming to support that DpdtpA induced EMT inhibition through a “fighting fire with fire” strategy (Figure S8, S9).

Taken together, DpdtpA-induced growth inhibition in the CT26 cells was ROS dependent. Mechanistic study revealed that DpdtpA was able to induce ferritinophagy that contributed to ROS production. Furthermore, DpdtpA was also able to inhibit EMT; the correlation analysis revealed that the ferritinophagic flux was a dominating factor in EMT process, attenuating the ferritinophagic flux resulted in EMT occurrence, while enhancing ferritinophagic flux favored to inhibit EMT (reversing EMT), indicating that NCOA4 played an important role in EMT process. In addition, DpdtpA induced EMT inhibition through producing massive ROS that were due to occurrence of ferritinophagy. However, the effect of ferritinophagic flux on EMT in different cell lines *in vivo* and *in vitro* requires more studies in the future due to the diversity of iron chelators in structure and complex interactions with biological molecules.

## 4. Materials and Methods

### 4.1. Materials

All chemicals used were analytical reagents (AR) grade. 3-(4,5-Dimethylthiazol-2-yl)-2,5-diphenyltetrazolium bromide (MTT), monodansylcadaverine (MDC), 3-methyladenine (3-MA), chloroquine, dichlorofluorescein (H_2_DCF-DA), desferoxamine (DFO), 4 ′,6-diamidino-2-phenylindole (DAPI), Roswell Park Memorial Institute (RPMI) 1640, and other chemicals were purchased from Sigma-Aldrich. Antibodies of vimentin (60330-1-lg, 10366-1-AP), LC3 (14600-1-AP), NCOA4 (E11-17114C), N-cadherin (22018-1-AP), and Gadph (E12-052) for Western blotting were obtained from Proteintech Group Inc. (Wuhan, China). Antibodies of E-cadherin (3195), ferritin (H chain, 3998S), secondary antibodies (fluorescence labeled for immunofluorescence, 8890S, 4412S), and cathepsin D were purchased from Cell Signaling Technology (USA). Ferritin antibody (SC-376594) for immunofluorescence was obtained from Santa Cruz Biotechnology (USA, Santa Cruz). NCOA4 antibody (HPA0512) for immunofluorescence was purchased from Atlas Antibody (Sweden). Secondary antibodies for Western blotting were obtained from EarthOx, LLC (San Francisco, USA).

### 4.2. Cytotoxicity Assay (MTT Assay)

The stock solution of DpdtpA (10 mM) was prepared in 70% DMSO and diluted to the required concentration with 70% DMSO. The CT26 cells were cultured in RPMI 1640 medium supplemented with 10% fetal calf serum (FCS) and antibiotics. The cells in the exponential phase were collected and seeded equivalently into a 96-well plate; next, the varied DpdtpA (or in the presence of NAC) was added after the cells adhered. Following 48 h incubation at 37°C in a humidified atmosphere of 5% CO_2_, 10 *μ*l MTT solution (5 mg/ml) was added and further incubated for 4 h; next, 100 *μ*l DMSO was added in each well to dissolve the formazan crystals after removing cell culture. The measurement of absorbance of the solution was performed on a microplate reader (MK3, Thermo Scientific) at 570 nm. Percent growth inhibition was defined as percent absorbance inhibition within appropriate absorbance in each cell line. The same assay was performed in triplet.

### 4.3. Flow Cytometric Analysis of Apoptosis and Cellular ROS

Cells were seeded into a 6-well plate and treated as described in the section of cytotoxicity assay. The cells were treated with different concentrations of the agent (0.78 and 1.56 *μ*M DpdtpA) for 24 h. Then, the cell culture was removed, following PBS washing, trypsin digestion; finally, the annexin V and propidium iodide (a kit from Dojindo Laboratories, Kumamoto, Japan) were added as recommended by the company. The stained cells were subjected to flow cytometer analysis (Becton-Dickinson, USA). Similar to apoptosis assay, the CT26 cells were resuspended in H_2_DCF-DA containing serum-free culture medium and incubated for 30 min. Next, after removing the H_2_DCF-DA contained medium by centrifugation, washing with PBS, finally resuspended the cells in PBS. The intracellular ROS assay was performed on a flow cytometer (Becton-Dickinson, USA).

### 4.4. Immunofluorescence Analysis

The CT26 cells were first cultured in a 6-well plate with cover glass overnight. Following DpdtpA treatment for 24 h, cells were first fixed with 4% paraformaldehyde in PBS for 15 min at 37°C and then permeabilized with 0.2% triton-X-100 in PBS for 10 min. After blocking with 1% BSA in PBS for 30 min, the cells were incubated with either ferritin (H chain, Santa Cruz Biotechnology), or combined with LC3 (or NCOA4 (Atlas Antibodies)), or vimentin combined with E-cadherin (Cell Signaling Technology) primary antibody based on the protocol recommended by the company; at 4°C, the plate was shaken overnight. Next, removing the primary antibodies and washing with PBS, the cells were further incubated with fluorescence-labeled secondary antibody for 3 h at room temperature. After removing the secondary antibody, the cells were further counterstained with DAPI. Finally, a confocal laser scanning microscope (Nikon eclipse Ts2, Japan) was used to visualize the cells; the representative cells were selected and photographed.

### 4.5. Assay of Lysosomal Membrane Permeability (LMP)

The alteration of LMP was assayed as previously described [30]. For detection of the acidic cellular, LysoTracker Red (Invitrogen, USA) was used, which emits bright red fluorescence in acidic vesicles, after treatment of the cells with the agent, LysoTracker Red (the concentration used as recommended), for a period of 30 min. Following PBS washing, the fluorescent micrographs were captured using an inverted fluorescence microscope (Nikon eclipse Ts2, Japan).

### 4.6. Western Blotting Analysis

The protocol for Western blotting was followed as described previously [30]; briefly, 1 × 10^7^ CT26 cells that treated with or without DpdtpA were scraped in lysis buffer (50 mM Tris-HCl, pH 8.0, 150 mM NaCl, 1.0% NP-40, 10% glycerol, and protease inhibitors) on ice for 30 min., followed by centrifugation at 14,000 × g. The clear supernatant was stored at -80°C. Protein concentration was determined using a colorimetric Bio-Rad DC protein assay using the MK3 microplate reader at 570 nm. Proteins (30 *μ*g) were separated on a 13~15% sodium dodecyl sulfate-polyacrylamide gel at 200 V for 3 h. The separated proteins were subsequently transferred onto a PVDF membrane at 60 V for 2 h. The membrane was washed with Tris-buffered saline (TBS) and then blocked for 2 h in TBS containing 0.1% Tween-20 and 5% nonfat skimmed milk. The membrane was incubated at 4°C overnight with the appropriate primary antibody. The membrane was then washed several times with TBST and subsequently incubated with the appropriate HRP-conjugated secondary antibody (1 : 2,000 in TBST) for 1 h at room temperature. Following washing with TBST, the protein bands were detected using a super sensitive ECL solution (Boster Biological Technology Co. Ltd.) and visualized using a Syngene G:BOX imager (Cambridge, United Kingdom). Quantifications of protein band intensities and fluorescence intensity were performed using ImageJ software.

### 4.7. Statistical Analysis

Data were analyzed with Prism 5.0 (GraphPad Software Inc., USA). Comparisons were made using a one-way analysis of variance or a two-tailed *t*-test. Results are presented as the mean ± SEM. A *p* value < 0.05 was considered statistically significant.

## Data Availability

The data used to support the findings of this study are included within the article.

## Supplementary Materials

Supplementary Materials Figure S1: NAC attenuated DpdtpA-induced growth inhibition of the CT26 cell. The procedure was similar to that in the MTT assay section except 24 h incubation. The experimental condition was as indicated (∗∗^,^ ## *p* < 0.05; one-way ANOVA). Figure S2: MG312 did not attenuate DpdtpA-induced ferritin degradation. The experiments were performed twice. Figure S3: quantification analyses of autophagy-related proteins. The results of quantification analysis of LC3-II and beclin were from two experiments (^∗∗^ *p* < 0.05, ^∗∗∗^ *p* < 0.01 compared to DMSO; ^###^ *p* < 0.01 compared to 1.56 *μ*M treated group). Figure S4: Alteration of iron abundance when the cells were subjected to DpdtpA treatment or combination with 3-MA (^∗∗∗^ *p* < 0.01). Figure S5: MDC staining for determination of autophagosomes induced by DpdtpA. The condition was as indicated. Figure S6: TGF-*β*1 induced cellular alteration in morphology. (A) The treatment without TGF-*β*1 and (B) with TGF-*β*1. Figure S7: NCOA4 plays a role in EMT transformation. (A) Western blotting analyses of EMT-related and ferritinophagy proteins; (B) quantification analyses of E-cadherin, vimentin, ferritin, NCOA4, and ferritinophagic flux. The experiments were performed thrice (^∗∗^ *p* < 0.05 and ^∗∗∗^ *p* < 0.01). Figure S8: ROS production induced by DpdtpA was a time-dependent manner. The condition was indicated in the figure. Figure S9: ROS scavenger attenuated the ability of DpdtpA in EMT reversal. (A) The alterations in EMT-related markers when the CT26 cells treated by DpdtpA in the absence or presence of NAC; (B) quantification analysis based on (A). The condition was indicated in the figure. The experiments were performed thrice (^∗∗∗^ *p* < 0.01)." Also, please enclose "^∗∗∗^ *p* < 0.01" in a math environment.
