# Supplementary material for: Ferritinophagic Flux Activation in CT26 Cells Contributed to EMT Inhibition Induced by a Novel Iron Chelator, DpdtpA
Source: Oxid Med Cell Longev. 2019 Jun 20;2019:8753413. doi: 10.1155/2019/8753413 (PMC6610730; doi:10.1155/2019/8753413)
Supplement: Supplementary Materials — Figure S1: NAC attenuated DpdtpA-induced growth inhibition of the CT26 cell. The procedure was similar to that in the MTT assay section except 24 h incubation. The experimental condition was as indicated (∗∗, ## p < 0.05; one-way ANOVA). Figure S2: MG312 did not attenuate DpdtpA-induced ferritin degradation. The experiments were performed twice. Figure S3: quantification analyses of autophagy-related proteins. The results of quantification analysis of LC3-II and beclin were from two experiments (∗∗ p < 0.05, ∗∗∗ p < 0.01 compared to DMSO; ### p < 0.01 compared to 1.56 μM treated group). Figure S4: Alteration of iron abundance when the cells were subjected to DpdtpA treatment or combination with 3-MA (∗∗∗ p < 0.01). Figure S5: MDC staining for determination of autophagosomes induced by DpdtpA. The condition was as indicated. Figure S6: TGF-β1 induced cellular alteration in morphology. (A) The treatment without TGF-β1 and (B) with TGF-β1. Figure S7: NCOA4 plays a role in EMT transformation. (A) Western blotting analyses of EMT-related and ferritinophagy proteins; (B) quantification analyses of E-cadherin, vimentin, ferritin, NCOA4, and ferritinophagic flux. The experiments were performed thrice (∗∗ p < 0.05 and ∗∗∗ p < 0.01). Figure S8: ROS production induced by DpdtpA was a time-dependent manner. The condition was indicated in the figure. Figure S9: ROS scavenger attenuated the ability of DpdtpA in EMT reversal. (A) The alterations in EMT-related markers when the CT26 cells treated by DpdtpA in the absence or presence of NAC; (B) quantification analysis based on (A). The condition was indicated in the figure. The experiments were performed thrice (∗∗∗ p < 0.01)." Also, please enclose "∗∗∗ p < 0.01" in a math environment. [file 8753413.f1.docx]

**Supplementary Materials**

**DpdtpA induced growth inhibition was ROS dependent**

DpdtpA induced growth inhibition involved ROS production, whether there was any correlation between the two events. To determine the possible relationship, a ROS scavenger, NAC was used in the proliferation assay. To minimize the effect of NAC on growth of the CT26 cell, the cells were treated by varied concentration of NAC, there was no obvious growth inhibition between 0~0.3 mM, thus 0.15 mM was chosen. As shown in Figure S1, the addition of NAC could significantly attenuate the growth inhibition induced by DpdtpA. The growth inhibition induced by DpdtpA or combined with NAC had statistical significance (p<0.05), indicating that DpdtpA induced growth inhibition was ROS dependent.


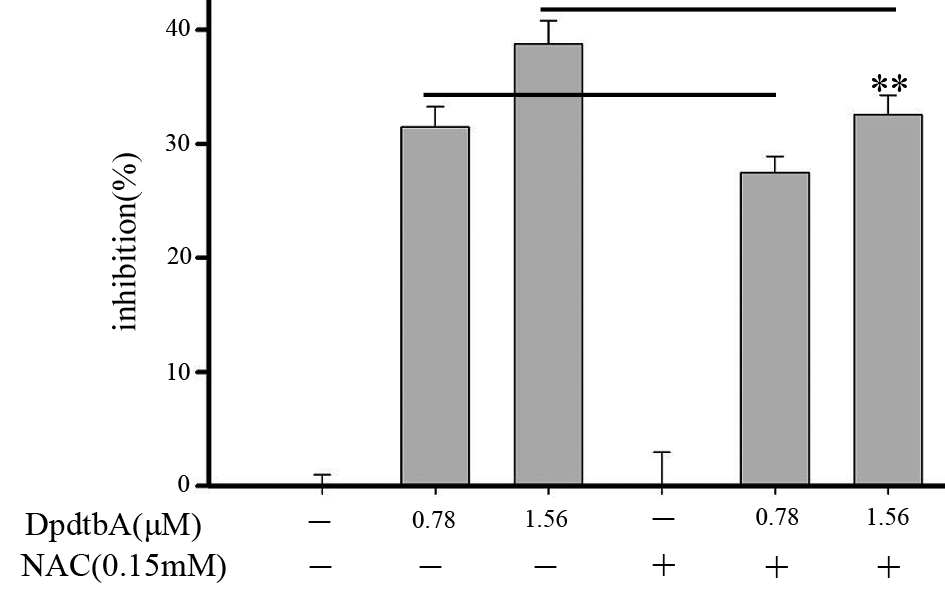


**Figure S1**: NAC attenuated DpdtbA induced growth inhibition of CT26 cell. The procedure was similar to that in MTT assay section except 24 h incubation. The experimental condition was as indicated. (**, ^##^, p<0.05; one-way ANOVA )

**DpdtpA induced ferritin degradation did not occur in proteasomes**

As well documented, ferritin degradation generally occurs either in proteasomes or lysosomes, thus we determined whether it degraded in proteasomes through ubiquitination. To this end, MG132, a proteasomes inhibitor was employed, as shown in Figure S2, DpdtpA could induce ferritin degradation, but the addition of MG132 did not attenuate ferritin degradation, indicating that DpdtpA induced ferritin degradation did not occur in proteasomes.


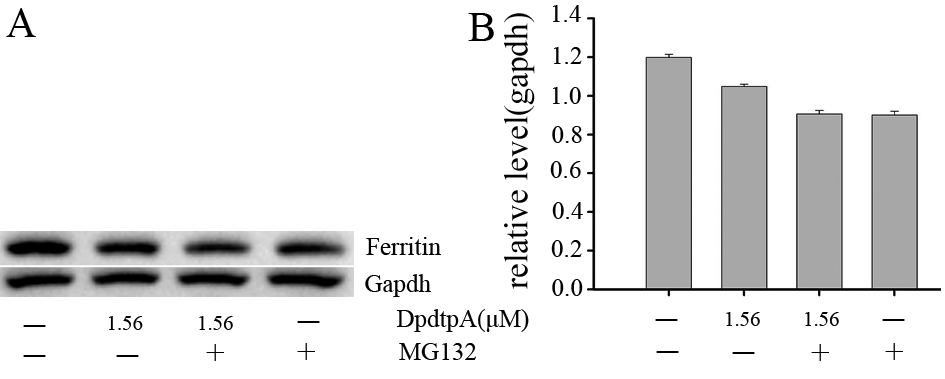


**Figure S2**: MG312 did not attenuate DpdtpA induced ferritin degradation. The experiments were performed twice.

**DpdtpA induced an occurrence of autophagy in CT26 cells**

Concomitant to the loss of ferritin, the DpdtpA induced an occurrence of autophagy due to upregulation of LC3-II and Beclin（Figure 5），the quantification analysis of autophagy-related proteins is given in Figure S3.

**
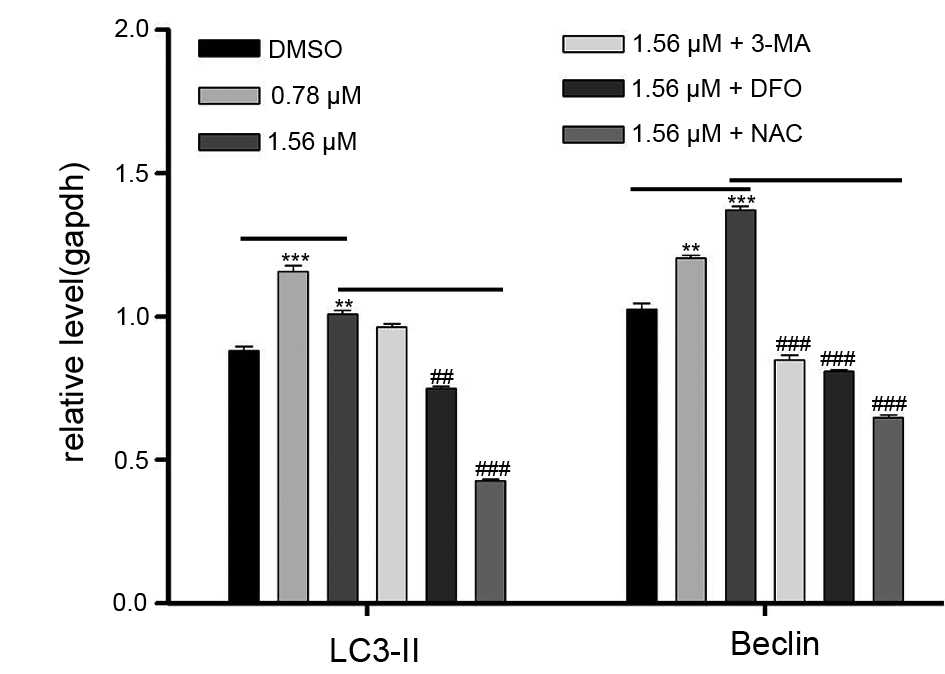
**

Figure S3: Quantification analyses of autophagy- related proteins. The results of quantification analysis of LC3-II and beclin were from two experiments. (**P<0.05, *** P<0.01 compared to DMSO; ^###^ P<0.01 compared to 1.56 μM treated group)

.

**DpdtpA induced decrease of total iron abundance was relative to autophagy**

The total iron content of CT26 was determined by atomic absorption spectroscopy (AAS) (Persee, Beijing, China, model TAS-900) based on a method reported previously with some modification [1]. The cultured cells were washed three times with a HEPES-buffered solution (154 mmol/L NaCl, 10 mM HEPES, pH 7.4). Cells were lysed by addition of HNO_3_ with heating, and the iron content was determined by AAS. A cell-free sample was prepared in same way and used as a blank.


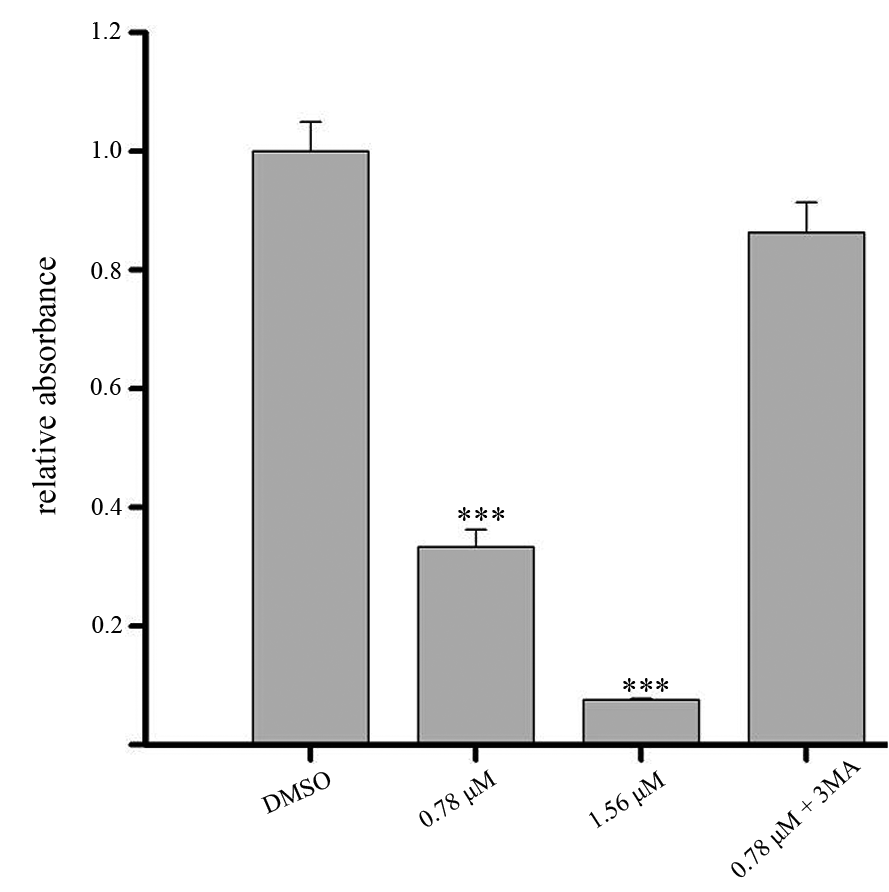


**Figure S4**: Alteration of iron abundance when the cells were subjected to DpdtpA treatment or combination with 3-MA. (***, p<0.01).

**Microscopic analyses of autophagic vacuoles**

CT26 Cells were seeded into a 6-well plate and treated as described above in cell viability assay. The cells were treated with either DpdtpA alone (0.78 or 1.56 μM) or combination with DFO (100 μM), 3-MA (5 mM) and NAC (1.5 mM)) for 24 h, respectively. Then the cell culture was removed, following PBS washing, trypsin digestion, finally the MDC (50 μM) were added as described previously [2]. The stained cells were observed on a fluorescence microscopy (Nikon eclipse Ts2, Japan).


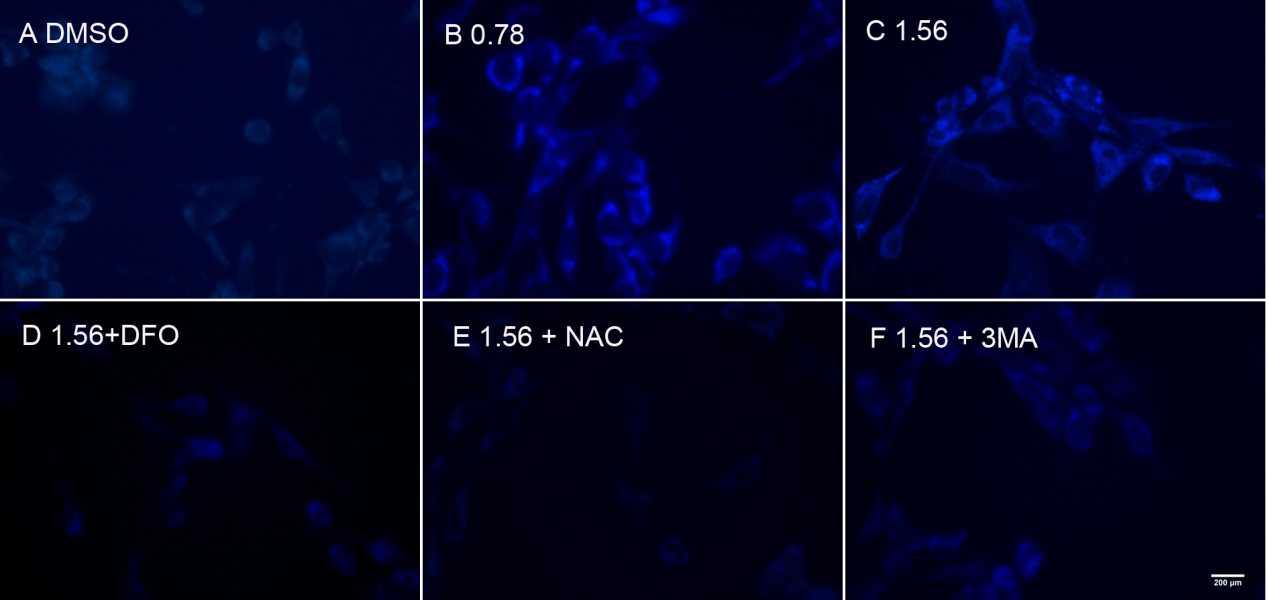


Figure S5: MDC staining for determination of autophagosomes induced by DpdtpA. The condition was as indicated.

**TGF-β1 induced cellular alteration in morphology**

To validate that DpdtpA’s capacity in EMT reversal, an EMT model required to be established. To this end, TGF-β1, a powerful EMT inducer was used. As shown in Figure S6, the CT26 cells were spindle-shaped, fibroblast-like after TGF-β1 treatment, indicating that CT26 cells were undergoing EMT.


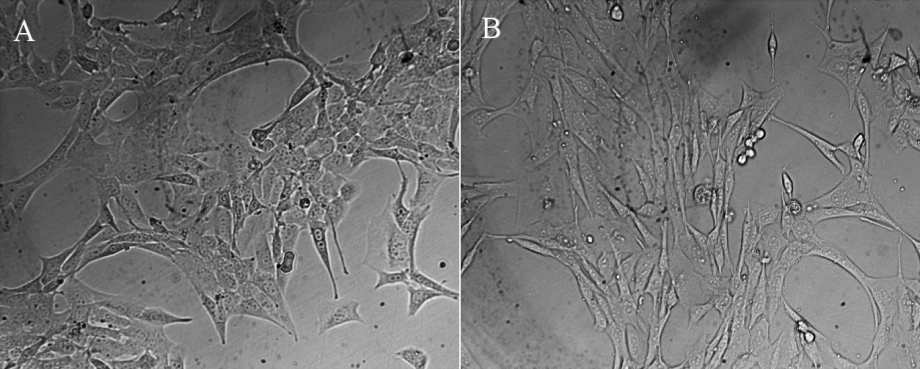


Figure S6: TGF-β1 induced cellular alteration in morphology. (A) the treatment without TGF-β1 and (B) with TGF-β1.

**Attenuating ferritinophagic flux by knocking down of NCOA4 could promote EMT**

To further confirm that the NCOA4 involved EMT inhibition induced by DpdtpA, the small-interfering RNA (si-RNA) was used to knockdown NCOA4. Briefly, after removing culture and washing with PBS, CT26 cells (1×10^6^) were transfected with 100 pmol of siRNA using Lipofectamine™ Stem Transfection Reagent (Invitrogen, USA) for 12 h following a protocol recommended by the manufacturer. Next the DpdtpA was added to the cells with complete medium for 24 h incubation at 37℃ in a humidified atmosphere of 5% CO_2_. The Western blotting analysis is shown in Figure S7, DpdtpA induced both EMT inhibition (E-cadherin increased, vimentin decreased) and ferritinophagy, and more interestingly, downregulation of NCOA4 clearly attenuated ferritinophagic flux, led to mesenchymal transformation, indicating that NCOA4 played role in EMT, which was firstly observed during EMT process.

**
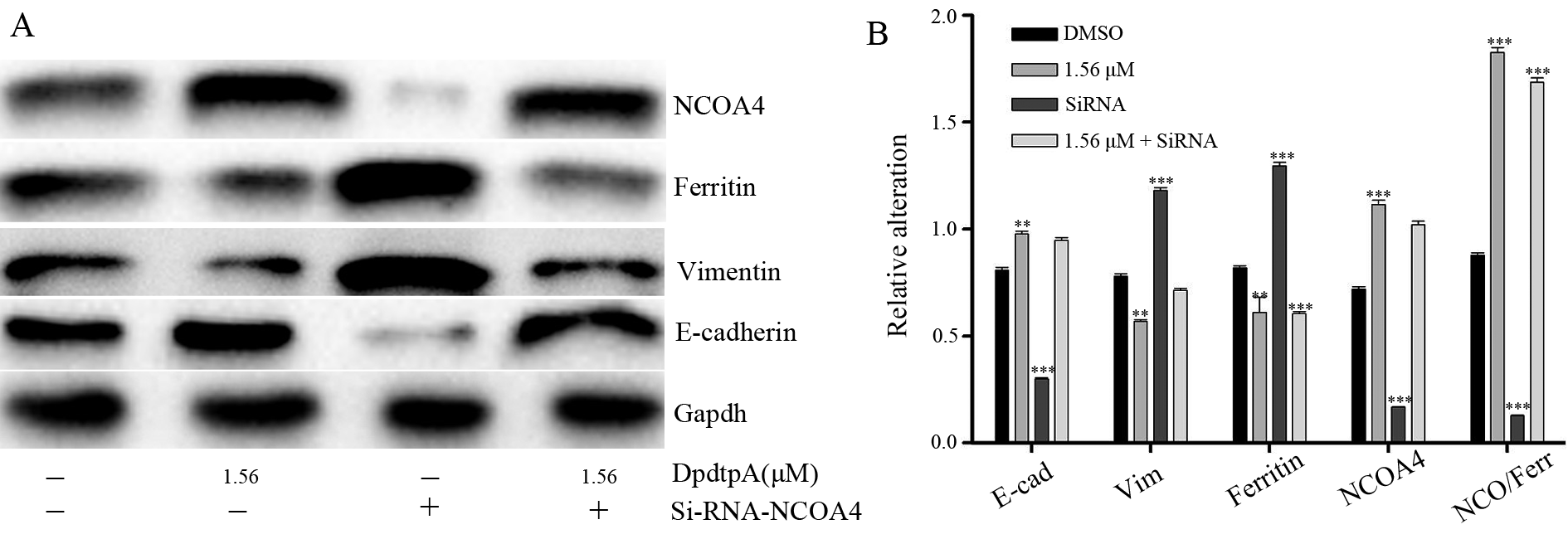
**

Figure S7: NCOA4 plays role in EMT transformation. (A) Western blotting analyses of EMT-related and ferritinophagy proteins; (B) quantification analyses of E-cadherin, vimentin, ferritin, NCOA4 and ferritinophagic flux. The experiments were performed thrice (^**^p<0.05 and ^***^ p<0.01).

**DpdtpA inhibited TGF- β1induced EMT was through generating massive ROS**

The ROS production at different time was assay by DCF stain as described in main text. As shown in Figure S8, a fluctuating ROS was observed in TGF-β1 treated CT26 cells, while DpdtpA induced a constant ROS production, the maximum of ROS was achieved at 24 h. Similar trend was observed in TGF- β1 combined with DpdtpA treatment. This hinted that DpdtpA suppressed TGF- β1 induced EMT might be through generating massive ROS, i.e., “fighting fire with fire” strategy (24 h). To validate this hypothesis, a ROS scavenger, NAC was introduced to this experiment, the levels of EMT-related proteins were determined by Western blotting. As shown in Figure S9, the DpdtpA caused significant increase of epithelial marker, E-cadherin, contrarily the mesenchymal markers, vimentin and N-cadherin were downregulated. Interestingly, the addition of NAC significantly attenuated the effect of DpdtpA on EMT, seeming to support that DpdtpA suppressed TGF- β1 induced EMT was achieved through “fighting fire with fire” strategy.


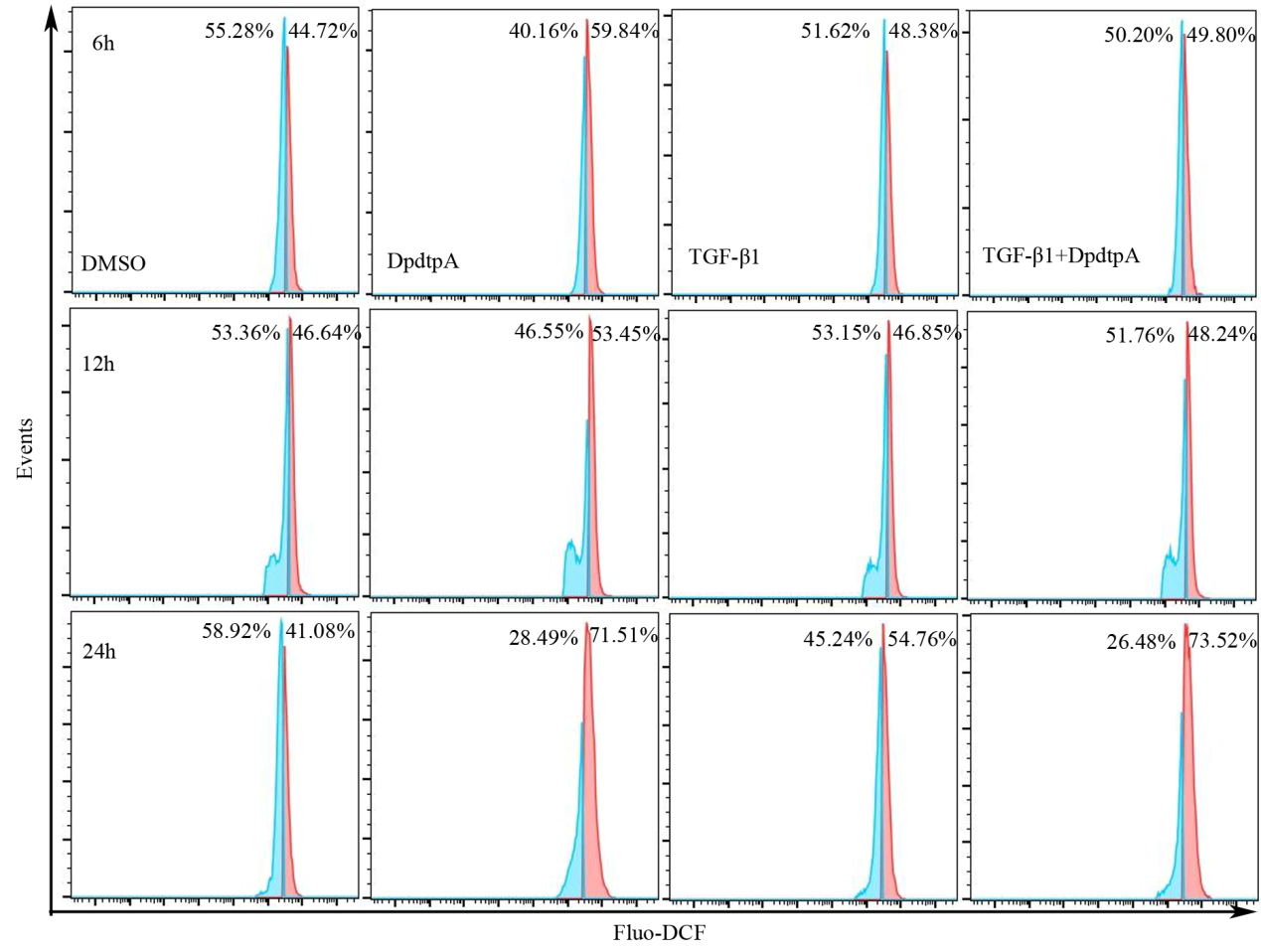


Figure S8: ROS production induced by DpdtpA was a time dependent manner. The condition was indicated in the figure.


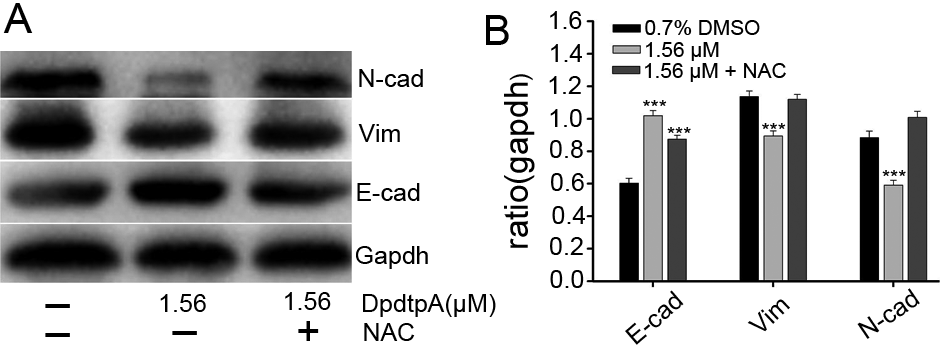


Figure S9: ROS scavenger attenuated the ability of DpdtpA in EMT reversal. (A) The alterations in EMT-related markers when the CT26 cells treated by DpdtpA in the absence or presence of NAC; (B) quantification analysis based on (A). The condition was indicated in the figure. The experiments were performed thrice. (^***^P<0.01).

**Reference**

1. Jan Riemer, Hans Hermann Hoepken, Hania Czerwinsk, Stephen R. Robinson, and Ralf Dringen, “Colorimetric ferrozine-based assay for the quantitation of iron in cultured cells,” Analytical Biochemistry 331 (2004) 370–375.
2. Xingshuang Guo, Yun Fu, Zuo Wang, et al., “Di-2-pyridylhydrazone dithiocarbamate butyric acid ester exerted its proliferative inhibition against gastric cell via ROS-Mediated apoptosis and autophagy”, Oxidative Medicine and Cellular Longevity, Volume 2018, Article ID 4950705, 11 pages.
